# Silver diaquacobalt(II) *catena*-borodiphosphate(V) hydrate, (Ag_0.79_Co_0.11_)Co(H_2_O)_2_[BP_2_O_8_]·0.67H_2_O

**DOI:** 10.1107/S1600536811052020

**Published:** 2011-12-07

**Authors:** Hafid Zouihri, Mohamed Saadi, Boujemaa Jaber, Lehcen El Ammari

**Affiliations:** a Centre National pour la Recherche Scientifique et Technique, Division UATRS, Angle Allal AlFassi et Avenue des FAR, Hay Ryad, BP 8027, Rabat, Morocco; b Laboratoire de Chimie du Solide Appliquée, Faculté des Sciences, Université Mohammed V-Agdal, Avenue Ibn Batouta, BP 1014, Rabat, Morocco

## Abstract

The structure of the title compound, (Ag_0.79_Co_0.11_)Co(H_2_O)_2_[BP_2_O_8_]·0.67H_2_O is isotypic to that of its recently published counterparts AgMg(H_2_O)_2_[BP_2_O_8_]·H_2_O and (Ag_0.57_Ni_0.22_)Ni(H_2_O)_2_[BP_2_O_8_]·0.67H_2_O. It consists of infinite borophos­phate helical ribbons [BP_2_O_8_]^3−^, built up from alternate BO_4_ and PO_4_ tetra­hedra arranged around the 6_5_ screw axes. The vertex-sharing BO_4_ and PO_4_ tetra­hedra form a spiral ribbon of four-membred rings in which BO_4_ and PO_4_ groups alternate. The ribbons are connected through slightly distorted CoO_4_(H_2_O)_2_ octa­hedra whose four O atoms belong to the phosphate groups. The resulting three-dimensional framework is characterized by hexa­gonal channels running along [001] in which the remaining water mol­ecules are located. The main difference between the Mg-containing and the title structure lies in the filling ratio of Wyckoff positions 6*a* and 6*b* in the tunnels. The refinement of the occupancy rate of the site 6*a* shows that it is occupied by water at 67%, while the refinement of that of the site 6*b* shows that this site is partially occupied by 78.4% Ag and 10.8% Co, for a total of 82.2%. The structure is stabilized by O—H⋯O hydrogen bonds between water mol­ecules and O atoms that are part of the helices.

## Related literature

For the isotypic Mg and Ni analogues, see: Zouihri *et al.* (2011*a* ▶,*b* ▶); Menezes *et al.* (2008 ▶). For other similar borophosphates, see: Kniep *et al.* (1997 ▶, 1998 ▶); Ewald *et al.* (2007 ▶); Lin *et al.* (2008 ▶). For ionic radii, see: Shannon (1976 ▶).

## Experimental

### 

#### Crystal data

(Ag_0.79_Co_0.11_)Co(H_2_O)_2_[BP_2_O_8_]·0.67H_2_O
*M* *_r_* = 398.78
Hexagonal, 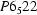

*a* = 9.4321 (11) Å
*c* = 15.750 (4) Å
*V* = 1213.5 (4) Å^3^
*Z* = 6
Mo *K*α radiation
μ = 4.63 mm^−1^
*T* = 296 K
0.16 × 0.12 × 0.10 mm

#### Data collection

Bruker APEXII CCD detector diffractometer
Absorption correction: multi-scan (*SADABS*; Sheldrick, 1999 ▶) *T*_min_ = 0.519, *T*_max_ = 0.630
7995 measured reflections
974 independent reflections
748 reflections with *I* > 2σ(*I*)
*R*_int_ = 0.096

#### Refinement

*R*[*F* ^2^ > 2σ(*F* ^2^)] = 0.040
*wR*(*F* ^2^) = 0.093
*S* = 1.07
974 reflections
77 parameters
1 restraint
H-atom parameters constrained
Δρ_max_ = 0.83 e Å^−3^
Δρ_min_ = −0.54 e Å^−3^
Absolute structure: Flack (1983 ▶), 340 Friedel pairs
Flack parameter: −0.05 (5)

### 

Data collection: *APEX2* (Bruker, 2005 ▶); cell refinement: *SAINT* (Bruker, 2005 ▶); data reduction: *SAINT*; program(s) used to solve structure: *SHELXS97* (Sheldrick, 2008 ▶); program(s) used to refine structure: *SHELXL97* (Sheldrick, 2008 ▶); molecular graphics: *ORTEP-3 for Windows* (Farrugia, 1997 ▶) and *DIAMOND* (Brandenburg, 2006 ▶); software used to prepare material for publication: *WinGX* (Farrugia, 1999 ▶).

## Supplementary Material

Crystal structure: contains datablock(s) I, global. DOI: 10.1107/S1600536811052020/ru2019sup1.cif

Structure factors: contains datablock(s) I. DOI: 10.1107/S1600536811052020/ru2019Isup2.hkl

Additional supplementary materials:  crystallographic information; 3D view; checkCIF report

## Figures and Tables

**Table 1:** Hydrogen-bond geometry (Å, °)

*D*—H⋯*A*	*D*—H	H⋯*A*	*D*⋯*A*	*D*—H⋯*A*
O5—H5*A*⋯O4^i^	0.86	1.89	2.748 (6)	178
O5—H5*B*⋯O2	0.86	1.90	2.750 (6)	171
O6—H6*A*⋯O5^ii^	0.88	2.44	3.139 (11)	137

Symmetry codes: (i) 
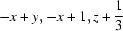
; (ii) 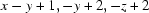.

**Fig. 1.**
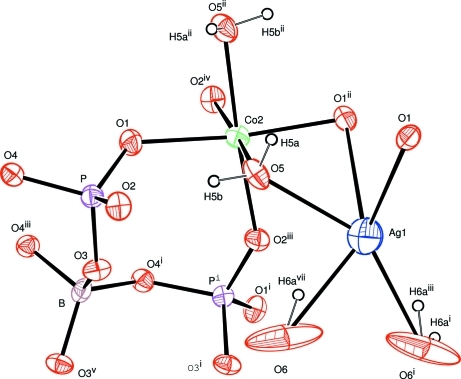
Partial plot of (Ag0.79Co0.11)Co(H2O)2[BP2O8],0.67(H2O) crystal structure showing polyhedra linkage. Displacement ellipsoids are drawn at the 50% probability level. Symmetry codes: (i) -y + 1, -x + 1, -z + 13/6; (ii) y - 1, -x + y, z + 1/6; (iii) y - 1, x, -z + 5/3; (iv) x, x-y + 1, -z + 11/6; (v) -x + y - 1, y, -z + 3/2; (vi) -x, -x + y, -z + 4/3; (vii) y, x + 1, -z + 5/3; (viii) x-y + 1, -y + 2, -z + 2.

**Fig. 2.**
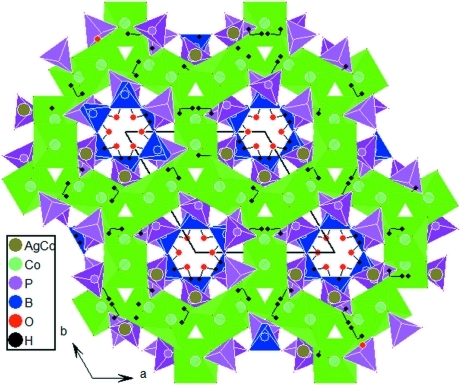
Projection view of the (Ag0.79Co0.11)Co(H2O)2[BP2O8],0.67(H2O) framework structure showing tunnel running along c direction where water molecules are located.

## supplementary crystallographic information

### Comment

A large group of borophosphates is known with a molar ratio of B:*P* = 1:2 and helical structure type, which consist of loop branched chain anions built from tetrahedral BO_4_ and PO_4_ units (Kniep *et al.*, (1998); Menezes *et al.*,(2008) and Lin *et al.* (2008)).

The aim of this work is the synthesis and the crystal structure of a new borophosphate-hydrate (Ag_0.79_Co_0.11_)Co(H_2_O)_2_[BP_2_O_8_],0.67(H_2_O), which is isotypic to the analogue nickel and magnesium borophosphates (Ag_0.57_Ni_0.22_)Ni(H_2_O)_2_[BP_2_O_8_],0.67(H_2_O); AgMg(H_2_O)_2_[BP_2_O_8_],H_2_O recently published (Zouihri *et al.*, 2011*a*, 2011*b*)) and to *M*^(I)^*M*^(II)^(H_2_O)_2_[BP_2_O_8_] H_2_O (*M*^(I)^=Li, Na, K, NH_4_^+^; *M*^(II)^= Mg, Mn, Fe, Co, Ni, Cu, Zn, Cd) (Kniep *et al.*, (1997) and Ewald *et al.*, (2007)).

The anionic partial structure of the title compound contains one–dimensional infinite helices, [BP_2_O_8_]^3-^, which are wound around 6_5_ axis. It is built up from alternate borate (BO_4_) and phosphate (PO_4_) tetrahedra, forming a spiral ribbon. The Co^2+^ cations have a slightly distorted octahedral oxygen coordination by four oxygen atoms from the phosphate anion and by two from water molecules as shown in Fig.1. The ribbons are interconnected through CoO_4_(H_2_O)_2_ octahedra. The resulting 3-D framework shows hexagonal tunnels running along *c* direction where water molecules are located (Fig.2).

The main difference between the structure of this compound and that of his counterpart AgMg(H_2_O)_2_[BP_2_O_8_],H_2_O lies in the filling ratio of the Wyckoff positions *6a* and *6 b* in tunnels. Indeed, in this work, the refinement of the occupancy rate of the sites *6a* and *6 b* (space group P6_5_22) shows that the first is occupied by water at 67% and the second is partially occupied by 78.4% of Ag and 10.8% of Co for a total of 82.2%. Note that in this case, the sum of the occupancie rate is restrained to fit the charge balance. While in the case of AgMg(H_2_O)_2_[BP_2_O_8_],H_2_O structure these two sites are completely occupied by H_2_O and Ag^+^ respectively.

It is interesting to compare the lattice parameters and volumes of title compound (Table 1) with some borophosphates of this family like AgMg(H_2_O)_2_[BP_2_O_8_],H_2_O (*a* = 9.4577 (4) Å, *c* = 15.830 (2)Å and *V* = 1226.4 (2)Å_3_) and (Ag_0.57_Ni_0.22_)Ni(H_2_O)_2_[BP_2_O_8_],0.67(H_2_O) (*a* = 9.3848 (6) Å, *c* = 15.841 (2)Å and *V* = 1208.3 (2)Å_3_). The ionic radii of Mg_II_, Co_II_, and Ni_II_ in the octahedral site are 0.72 Å, 0.74 Å and 0.69 Å, respectively (Shannon, 1976). The difference between these values is very small, therefore the filling rate of *6a* and *6 b* sites by (Ag_I_/ *M*_II_) and H_2_O, respectively, leads to the variation of the lattice parameters and volumes of these compounds. Indeed the obtained values for the title compound are between these of the two precedents borophosphates as expected.

The structure is stabilized by O—H···O hydrogen bonds between water molecules and O atoms that are part of the helices (Table 2).

### Experimental

The title borophosphate compound was hydrothermally synthesized at 453 °K for 7 days in a 25 ml Teflon-lined steel autoclave from the mixture of CoCO_3_, H_3_BO_3_, H_3_PO_4_ (85%), AgNO_3_ and 5 ml of distilled water in the molar ratio of 1:4:6:1:165. The reaction product was separated by filtration, washed with hot water and dried in air. The pink hexagonal bipyramid crystals obtained were up to 0.15 mm in length. Except for boron and hydrogen the presence of the elements were additionally confirmed by EDAX measurements. Indeed, the results of semi quantitative EDAX measurements are: Element, in At %: BK = 22.45; OK = 59.13; PK = 8.88; Ag *L* = 4.68, Co = 4.87 K. These values show a large excess of boron which is not surprising because the excess of boron comes from the synthesis of crystals.

### Refinement

The highest peak and the minimum peak in the difference map are at 0.89 Å and 0.98 Å respectively from Ag1 and P atoms. The O-bound H atom is initially located in a difference map and refined with O—H distance restraints of 0.86 (1). In a the last cycle there is refined in the riding model approximation with *U*_iso_(H) set to 1.5*U*_eq_(O). The 340 Friedel opposite reflections are not merged.

### Crystal data

**Table:**

(Ag_0.79_Co_0.11_)Co(H_2_O)_2_[BP_2_O_8_]·0.67H_2_O	*D*_x_ = 3.274 Mg m^−^^3^
*M**_r_* = 398.78	Mo *K*α radiation, λ = 0.71073 Å
Hexagonal, *P*6_5_22	Cell parameters from 974 reflections
Hall symbol: P 65 2 ( 0 0 1)	θ = 2.8–27.9°
*a* = 9.4321 (11) Å	µ = 4.63 mm^−^^1^
*c* = 15.750 (4) Å	*T* = 296 K
*V* = 1213.5 (4) Å^3^	Prism, pink
*Z* = 6	0.16 × 0.12 × 0.10 mm
*F*(000) = 1155	

### Data collection

**Table:**

Bruker APEXII CCD detector diffractometer	974 independent reflections
Radiation source: fine-focus sealed tube	748 reflections with *I* > 2σ(*I*)
graphite	*R*_int_ = 0.096
ω and φ scans	θ_max_ = 27.9°, θ_min_ = 2.8°
Absorption correction: multi-scan (*SADABS*; Sheldrick, 1999)	*h* = −12→11
*T*_min_ = 0.519, *T*_max_ = 0.630	*k* = −10→12
7995 measured reflections	*l* = −15→20

### Refinement

**Table:**

Refinement on *F*^2^	Secondary atom site location: difference Fourier map
Least-squares matrix: full	Hydrogen site location: difference Fourier map
*R*[*F*^2^ > 2σ(*F*^2^)] = 0.040	H-atom parameters constrained
*wR*(*F*^2^) = 0.093	*w* = 1/[σ^2^(*F*_o_^2^) + (0.0409*P*)^2^ + 1.3662*P*] where *P* = (*F*_o_^2^ + 2*F*_c_^2^)/3
*S* = 1.07	(Δ/σ)_max_ < 0.001
974 reflections	Δρ_max_ = 0.83 e Å^−^^3^
77 parameters	Δρ_min_ = −0.54 e Å^−^^3^
1 restraint	Absolute structure: Flack (1983), 340 Friedel pairs
Primary atom site location: structure-invariant direct methods	Flack parameter: −0.05 (5)

### Special details

**Table:**

Geometry. All e.s.d.'s (except the e.s.d. in the dihedral angle between two l.s. planes) are estimated using the full covariance matrix. The cell e.s.d.'s are taken into account individually in the estimation of e.s.d.'s in distances, angles and torsion angles; correlations between e.s.d.'s in cell parameters are only used when they are defined by crystal symmetry. An approximate (isotropic) treatment of cell e.s.d.'s is used for estimating e.s.d.'s involving l.s. planes.
Refinement. Refinement of *F*^2^ against all reflections. The weighted *R*-factor *wR* and goodness of fit *S* are based on *F*^2^, conventional *R*-factors *R* are based on *F*, with *F* set to zero for negative *F*^2^. The threshold expression of *F*^2^ > σ(*F*^2^) is used only for calculating *R*-factors(gt) *etc*. and is not relevant to the choice of reflections for refinement. *R*-factors based on *F*^2^ are statistically about twice as large as those based on *F*, and *R*- factors based on all data will be even larger.

### Fractional atomic coordinates and isotropic or equivalent isotropic displacement parameters (Å^2^)

**Table:**

	*x*	*y*	*z*	*U*_iso_*/*U*_eq_	Occ. (<1)
Ag1	0.18406 (7)	0.81594 (7)	1.0833	0.0491 (4)	0.784 (3)
Co1	0.18406 (7)	0.81594 (7)	1.0833	0.0491 (4)	0.1082 (16)
Co2	0.10332 (14)	0.55166 (7)	0.9167	0.0195 (3)	
P	0.16844 (18)	0.77958 (17)	0.75218 (11)	0.0176 (3)	
B	−0.1524 (6)	0.6952 (12)	0.7500	0.019 (2)	
O1	0.1357 (6)	0.6203 (5)	0.7906 (2)	0.0224 (11)	
O2	0.3146 (5)	0.9298 (5)	0.7861 (2)	0.0214 (10)	
O3	0.0203 (5)	0.8048 (5)	0.7667 (3)	0.0195 (10)	
O4	0.1833 (5)	0.7642 (5)	0.6541 (2)	0.0184 (9)	
O5	0.2914 (5)	0.8033 (5)	0.9461 (3)	0.0263 (11)	
H5A	0.3808	0.8058	0.9599	0.039*	
H5B	0.3104	0.8472	0.8965	0.039*	
O6	0.1183 (13)	1.0000	1.0000	0.099 (6)	0.67
H6A	0.2180	1.0790	0.9885	0.148*	0.67

### Atomic displacement parameters (Å^2^)

**Table:**

	*U*^11^	*U*^22^	*U*^33^	*U*^12^	*U*^13^	*U*^23^
Ag1	0.0534 (6)	0.0534 (6)	0.0438 (7)	0.0292 (6)	−0.0038 (5)	−0.0038 (5)
Co1	0.0534 (6)	0.0534 (6)	0.0438 (7)	0.0292 (6)	−0.0038 (5)	−0.0038 (5)
Co2	0.0187 (6)	0.0184 (5)	0.0214 (5)	0.0093 (3)	0.000	0.0008 (5)
P	0.0170 (8)	0.0188 (8)	0.0170 (7)	0.0090 (6)	0.0001 (7)	0.0000 (7)
B	0.021 (4)	0.022 (5)	0.014 (4)	0.011 (3)	0.004 (4)	0.000
O1	0.028 (3)	0.022 (2)	0.018 (2)	0.012 (2)	0.0026 (18)	0.0037 (17)
O2	0.017 (2)	0.020 (2)	0.022 (2)	0.006 (2)	−0.0012 (17)	0.0000 (18)
O3	0.014 (2)	0.016 (2)	0.027 (2)	0.0063 (18)	−0.0020 (17)	−0.0056 (18)
O4	0.019 (2)	0.018 (2)	0.019 (2)	0.0094 (19)	−0.0011 (16)	0.0008 (16)
O5	0.022 (2)	0.026 (3)	0.027 (2)	0.009 (2)	−0.0065 (18)	0.0060 (19)
O6	0.032 (6)	0.075 (11)	0.204 (17)	0.038 (6)	−0.043 (6)	−0.085 (12)

### Geometric parameters (Å, °)

**Table:**

Ag1—O5^i^	2.414 (4)	P—O3	1.547 (4)
Ag1—O5	2.414 (4)	P—O4	1.564 (4)
Ag1—O6^i^	2.489 (8)	B—O3^v^	1.452 (7)
Ag1—O6	2.490 (8)	B—O3	1.452 (7)
Co2—O1	2.063 (4)	B—O4^iii^	1.475 (7)
Co2—O1^ii^	2.063 (4)	B—O4^vi^	1.475 (7)
Co2—O2^iii^	2.084 (4)	O2—Co2^vii^	2.084 (4)
Co2—O2^iv^	2.084 (4)	O4—B^vi^	1.475 (7)
Co2—O5	2.188 (4)	O5—H5A	0.8601
Co2—O5^ii^	2.188 (4)	O5—H5B	0.8600
P—O2	1.497 (4)	O6—Ag1^viii^	2.490 (8)
P—O1	1.502 (4)	O6—H6A	0.8785
			
O5^i^—Ag1—O5	132.1 (2)	O1—P—O3	110.2 (3)
O5^i^—Ag1—O6^i^	79.6 (2)	O2—P—O4	111.0 (2)
O5—Ag1—O6^i^	147.77 (18)	O1—P—O4	106.7 (2)
O5^i^—Ag1—O6	147.77 (18)	O3—P—O4	106.8 (2)
O5—Ag1—O6	79.6 (2)	O3^v^—B—O3	103.8 (7)
O6^i^—Ag1—O6	69.9 (4)	O3^v^—B—O4^iii^	113.5 (2)
O1—Co2—O1^ii^	165.3 (3)	O3—B—O4^iii^	112.5 (2)
O1—Co2—O2^iii^	100.77 (16)	O3^v^—B—O4^vi^	112.5 (2)
O1^ii^—Co2—O2^iii^	89.30 (16)	O3—B—O4^vi^	113.5 (2)
O1—Co2—O2^iv^	89.30 (16)	O4^iii^—B—O4^vi^	101.5 (7)
O1^ii^—Co2—O2^iv^	100.77 (17)	P—O1—Co2	128.7 (3)
O2^iii^—Co2—O2^iv^	94.3 (3)	P—O2—Co2^vii^	139.9 (3)
O1—Co2—O5	87.21 (16)	B—O3—P	129.9 (4)
O1^ii^—Co2—O5	82.44 (16)	B^vi^—O4—P	130.2 (4)
O2^iii^—Co2—O5	87.56 (18)	Co2—O5—Ag1	96.38 (15)
O2^iv^—Co2—O5	176.30 (15)	Co2—O5—H5A	109.6
O1—Co2—O5^ii^	82.44 (16)	Ag1—O5—H5A	101.7
O1^ii^—Co2—O5^ii^	87.21 (16)	Co2—O5—H5B	101.0
O2^iii^—Co2—O5^ii^	176.31 (15)	Ag1—O5—H5B	141.3
O2^iv^—Co2—O5^ii^	87.56 (18)	H5A—O5—H5B	104.5
O5—Co2—O5^ii^	90.8 (3)	Ag1—O6—Ag1^viii^	106.6 (5)
O2—P—O1	115.7 (3)	Ag1—O6—H6A	99.5
O2—P—O3	106.1 (3)	Ag1^viii^—O6—H6A	20.2

Symmetry codes: (i) −*y*+1, −*x*+1, −*z*+13/6; (ii) *x*, *x*−*y*+1, −*z*+11/6; (iii) *y*−1, −*x*+*y*, *z*+1/6; (iv) *y*−1, *x*, −*z*+5/3; (v) −*x*+*y*−1, *y*, −*z*+3/2; (vi) −*x*, −*x*+*y*, −*z*+4/3; (vii) *y*, *x*+1, −*z*+5/3; (viii) *x*−*y*+1, −*y*+2, −*z*+2.

### Hydrogen-bond geometry (Å, °)

**Table:**

*D*—H···*A*	*D*—H	H···*A*	*D*···*A*	*D*—H···*A*
O5—H5A···O4^ix^	0.86	1.89	2.748 (6)	178.
O5—H5B···O2	0.86	1.90	2.750 (6)	171.
O6—H6A···O5^viii^	0.88	2.44	3.139 (11)	137.

Symmetry codes: (ix) −*x*+*y*, −*x*+1, *z*+1/3; (viii) *x*−*y*+1, −*y*+2, −*z*+2.
